# Copulation Activity, Sperm Production and Conidia Transfer in *Aedes aegypti* Males Contaminated by *Metarhizium anisopliae*: A Biological Control Prospect

**DOI:** 10.1371/journal.pntd.0004144

**Published:** 2015-10-16

**Authors:** Javier A. Garza-Hernández, Filiberto Reyes-Villanueva, Tanya L. Russell, Marieta A. H. Braks, Alberto M. Garcia-Munguia, Mario A. Rodríguez-Pérez

**Affiliations:** 1 Laboratorio de Biomedicina Molecular, Centro de Biotecnología Genómica, Instituto Politécnico Nacional, Tamaulipas, México; 2 Faculty of Medicine, Health and Molecular Sciences, James Cook University, Cairns, Queensland, Australia; 3 National Institute for Public Health and the Environment, Bilthoven, The Netherlands; 4 Centro de Ciencias Agropecuarias, Universidad Autónoma de Aguascalientes, carretera a la Posta, Jesús María, Aguascalientes, México; University of Perugia, ITALY

## Abstract

**Background:**

Dengue is the most prevalent arboviral disease transmitted by *Aedes aegypti* worldwide, whose chemical control is difficult, expensive, and of inconsistent efficacy. Releases of *Metarhizium anisopliae*—exposed *Ae*. *aegypti* males to disseminate conidia among female mosquitoes by mating represents a promising biological control approach against this important vector. A better understanding of fungus virulence and impact on reproductive parameters of *Ae*. *aegypti*, is need before testing auto-dissemination strategies.

**Methodology/Principal Findings:**

Mortality, mating competitiveness, sperm production, and the capacity to auto-disseminate the fungus to females up to the 5^th^copulation, were compared between *Aedes aegypti* males exposed to 5.96 x 10^7^ conidia per cm^2^ of *M*. *anisopliae* and uninfected males. Half (50%) of fungus-exposed males (FEMs) died within the first 4 days post-exposure (PE). FEMs required 34% more time to successively copulate with 5 females (165 ± 3 minutes) than uninfected males (109 ± 3 minutes). Additionally, fungus infection reduced the sperm production by 87% at 5 days PE. Some beneficial impacts were observed, FEMs were able to successfully compete with uninfected males in cages, inseminating an equivalent number of females (about 25%). Under semi-field conditions, the ability of FEMs to search for and inseminate females was also equivalent to uninfected males (both inseminating about 40% females); but for the remaining females that were not inseminated, evidence of tarsal contact (transfer of fluorescent dust) was significantly greater in FEMs compared to controls. The estimated conidia load of a female exposed on the 5^th^ copulation was 5,200 mL^-1^ which was sufficient to cause mortality.

**Conclusion/Significance:**

Our study is the first to demonstrate auto-dissemination of *M*. *anisopliae* through transfer of fungus from males to female *Ae*. *aegypti* during mating under semi-field conditions. Our results suggest that auto-dissemination studies using releases of FEMs inside households could successfully infect wild *Ae*. *aegypti* females, providing another viable biological control tool for this important the dengue vector.

## Author Summary

Dengue virus (four serotypes) is transmitted primarily by the mosquitoes *Aedes aegypti* and currently 2.5 billion people are in risk of being infected. The incidence of this neglected disease is increasing in developing countries where communities have not been able to effectively remove mosquito sources and their economies cannot afford the vector chemical control. Our study is collecting some of the baseline information necessary to evaluate a novel biological control strategy that would release *Ae*. *aegypti* males mosquitoes infected with the fungus *Metarhizium anisopliae*. These fungus-infected males transfer the fungus to female mosquitoes through leg contact during the mating process (This is called auto-dissemination). The fungus killed 50% of males within 4 days of being exposed. The fungus infection also increase the time that males need to mate with 5 females mosquitoes and reduced the sperm production by 87% 5 days after being infected. The ability of fungus-exposed males (FEMs) to find and mate with females in the laboratory or a small greenhouse was the same as for uninfected males. In particular, in the small greenhouse FEMs made more mating attempts without insemination than the uninfected males (more than twice the attempts). During both attempts and successful matings, the FEMS were able to transfer fungus to females confirming that auto-dissemination does occur. We also showed that the amount of fungus transferred to female, even after the 5^th^mating (about 10% of male’s conidia load) was sufficient to kill 50% of females within 3 days. These results indicate that there is potential for auto-dissemination of *M*. *anisopliae* from males to females as a dengue control tool.

## Introduction

*Aedes aegypti* is the principal vector of the four dengue (DENV) virus serotypes [1]. Although its control through larval source removal is effective, the only rapid but inconsistent way to interrupt epidemic transmission is by chemical insecticides [2,3]. The scarcity of natural enemies of *Ae*. *aegypti* [4,5] has led to promising research into biocontrol with entomopathogenic fungus. *Metarhizium anisopliae* and *Beauveria bassiana* have been examined by direct exposure of larvae to conidia (asexual, non-motile fungus spores) in water/oil, and through contact of resting adults on fungus-impregnated black clothes/nets [6–11]. Furthermore, *M*. *anisopliae* also reduces *Ae*. *aegypti* vectorial capacity by interfering with dengue virus replication; females co-infected with *M*. *anisopliae* and DENV-2 had lower viral loads in heads compared to females infected only with DENV-2 [12]. *Metarhizium anisopliae* is a hyphomycetous insect-pathogenic fungus of which the conidia infect insects by penetrating the cuticle. *Metarhizium* spp. are endemic worldwide and are not harmful to birds, fish, or mammals including humans [13]. Their pathogenicity/toxicity/allergenicity has been studied intensively representing only minimal risk to vertebrates, the environment and public health [14].

Auto-dissemination—the transfer of agents from infected organisms to others in a population—has been shown to occur through tarsal contact during copulation for entomopathogenic fungi associated with agricultural pests [15–17], but information for human disease vectors is far more limited. They include the transfer of *M*. *anisopliae* and *B*. *bassiana* between female and male *Glossina morsitans morsitans* that has been reported [18], and successful auto-dissemination of *M*. *anisopliae* from female to male *Anopheles gambiae* under laboratory conditions [19]. *M*. *anisopliae* conidia transfer from fungus-exposed males (FEMs) to female *Ae*. *aegypti* [20] could provide an additional tool for integrated dengue vector control programs through intradomicile releases of FEMs. The mating behavior of *Ae*. *aegypti* might favor auto-dissemination through the release of FEMs, because the polygamous males do not discriminate between virgin or mated female mosquitoes [21], although the insemination occurs only in the first 5 to 7 female mosquitoes [22]. Additionally, a small but potentially significant portion of females have been observed to mate multiple times within a 48-hour period under semi-field conditions [23]. In fact initial studies looking at conidia-transfer from FEMs to females in captivity have been promising [24]; a single *M*. *anisopliae*-exposed male after 48-h confinement with 30 female mosquitoes, infected the 85% of the females and killed the 50% in 7 days with a 90% sporulation and 99% fecundity reduction [20]. Prior to conducting a small-scale field trial of *M*. *anisopliae* auto-dissemination, it is critical to investigate numerous parameters; this paper contributes by extending baseline semi-field experiments [23] and evaluating the fungal effect on male sexual performance and the conidia transfer through successive copulations. Understanding any impact on the quantity of sexual encounters is relevant because the fungus is transferred from males to females by tarsal contact, which is the first step in the *Ae*. *aegypti* copulation process [25].

This study examined baseline parameters necessary to evaluate the potential of fungus auto-dissemination via the release of FEMs as a biocontrol tool, including the direct impact of fungal infection on male mosquito survival, mating success both under laboratory and semi-field conditions, copulation speed, sperm production, and finally the impact of conidia load on the FEMs to transfer lethal doses to exposed females.

## Material and Methods

### Mosquito colony

Survival and conidia-transfer experiments described below used 4–7 day-old, sugar-fed virgin male and female mosquitoes from an *Ae*. *aegypti* colony established in 2006 in Monterrey, NL, Mexico and maintained as described in Garcia-Munguía et. al. (2011). Experiments to evaluate sperm production in *Ae*. *aegypti* males exposed to *M*. *anisopliae* compared to controls were carried out in 3 day-old, sugar-fed virgin males.

### Conidia dose and mosquito infection

All reported experiments utilized the Ma-CBG-2 strain of *M*. *anisopliae* at an experimental conidial dose (ED) of 5.96 x 10^7^conidia cm^-2^ prepared as previously reported [20]. Briefly, the fungus was cultured in potato-dextrose-agar plates incubated at 25 ± 2°C for 20 days in the dark. The conidia yield was estimated by using a mixture of 0.5% Tween-20 and 0.5% Triton-X in 0.85% saline solution. Spore suspension was centrifuged at 3,500 rpm for 10 minutes diluted up to 1.6 x 10^8^ conidia mL^-1^ with a hemocytometer, and 5 ml were applied to a 2.5 μm pore, 8 cm diameter filter paper to set up the ED. Both treated and untreated filter papers were placed in chambers described previously [12], where mosquitoes were confined for 24-h. Insectary conditions were maintained at 25 ± 1°C, relative humidity (RH) of 80 ± 5%, and a photoperiod of 14:10-h L: D.

### Survival of *Ae*. *aegypti* males exposed to experimental conidial dose of *M*. *anisopliae*

Three replicate experiments were conducted, each compared 25 males mosquitoes exposed to ED treated filters with 25 male mosquitoes exposed to untreated control filters for 24-h. After exposure mosquitoes were transferred to separate 1-liter translucent plastic flasks covered with a mesh and a cotton pad soaked with 5% sucrose. The flasks were monitored daily for mortality. Cadavers were removed and twice submerged in 1% sodium hypochlorite, washed in distilled water, and placed in humid chambers for fungus sporulation.

### Effect of *M*. *anisopliae* infection on the mating activity of male *Ae*. *aegypti* under laboratory and small greenhouse conditions

The ability of FEMs to compete with uninfected males and successfully copulate with females was examined with the aid of fluorescent powders used to mark the male mosquitoes. Before application of the fluorescent powder, 10 male *Ae*. *aegypti* were transferred to a 180 ml paper cup covered with mesh and anaesthetized by exposure to 4°C for 5 minutes. The cup was placed in a plastic bag and each powder was applied by filling a syringe (5 ml with 0.6 × 25 mm needle) with fluorescent powder up to 0.5 ml. The syringe was inserted through the mesh at the top of the cup and with one gentle push the powder was blown out of the syringe marking the mosquitoes inside the cup [26]. The FEMs were marked with red powder and uninfected males were marked with yellow powder. Next, the mosquitoes were anaesthetized again by exposure to 4°C for 5 minutes for use in the following experiments.

#### Laboratory

**Fig 1:**
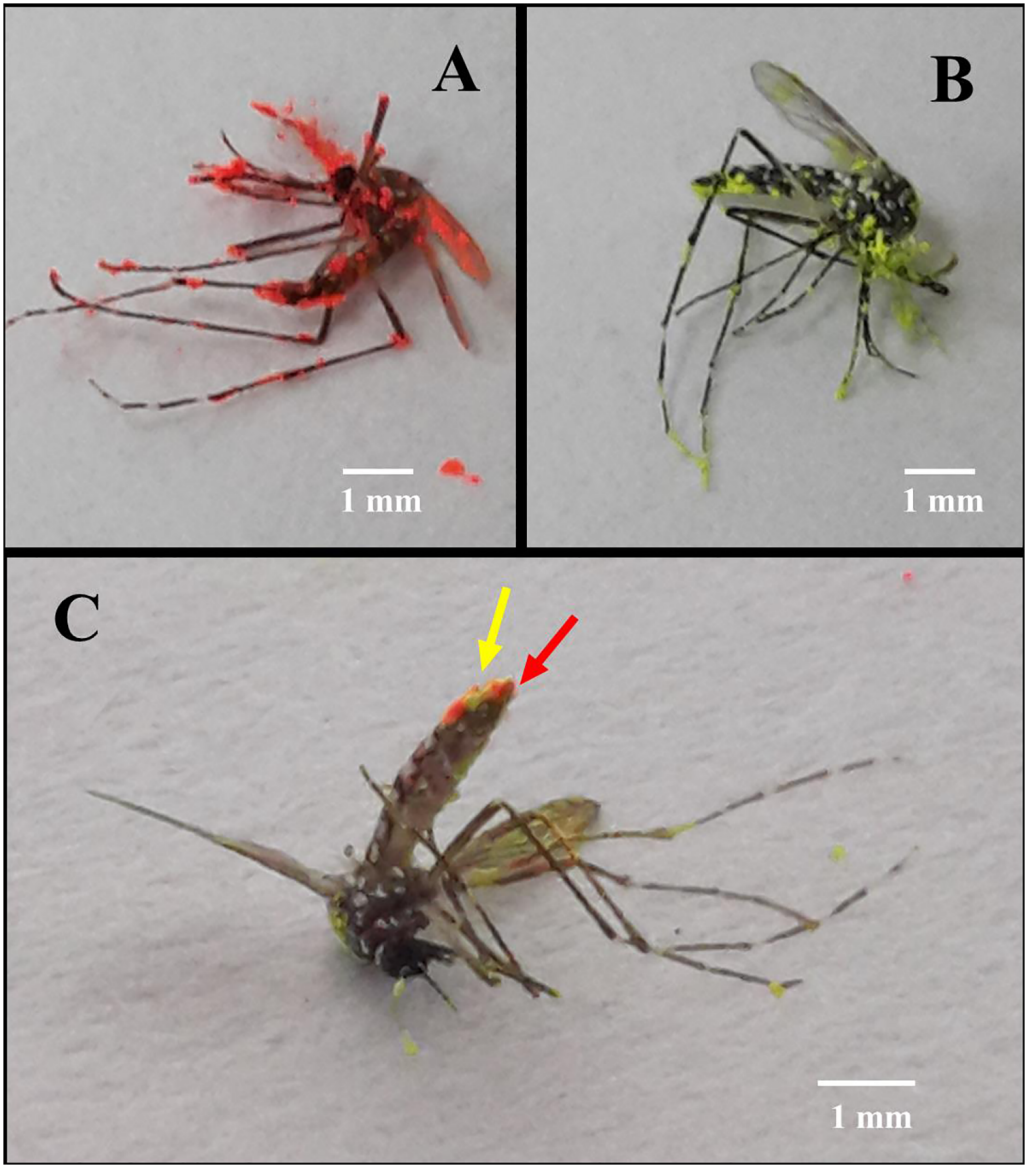
*Aedes aegypti* mosquito adults marked with fluorescent powders. A: *M*. *anisopliae*-exposed male marked with red fluorescent powder. B: Uninfected male marked with yellow powder. C: A female mosquito that was mated by both fungus-exposed male and uninfected one, showing spots of powder of both colors on the last abdominal segments (red and yellow arrows).

One FEM (marked with red dust), one uninfected male (marked with yellow dust) and 20 unmarked, uninseminated females were placed in a 1-liter plastic bottle, and confined together from 16:00 h to 19:00 h. At 19:00 h the mosquitoes were immobilized and the females were collected for dissection and removal of the spermathecae, which were examined for evidence of insemination. Before dissection the female mosquitoes were examined using a UV light for the presence of fluorescent powder transfer on to the last abdominal segment (Fig 1). Females were scored into the following four nominal response variable (RV) categories: Inseminated by FEM (red powder), inseminated by uninfected male (yellow powder), inseminated and encountered by both FEM and uninfected males (both red and yellow powder), and uninseminated females not contacted by any male (no powder). Ten replicates were carried out on different days all with a mixture of mosquitoes 4–7 days old.

#### Semi-field experiments

**Fig 2:**
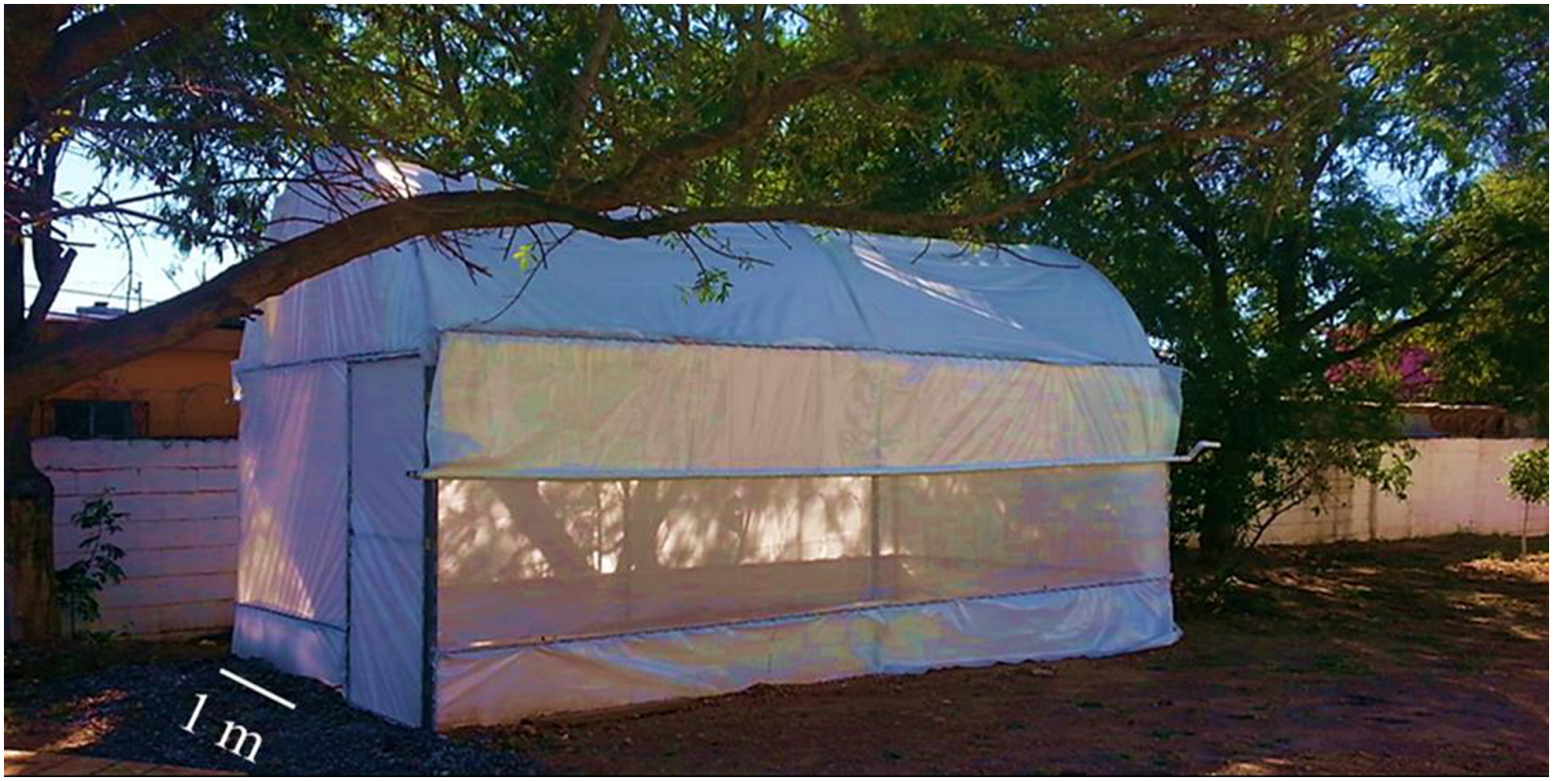
Greenhouse to evaluate copulation activity of *M*. *anisopliae*-exposed and uninfected *Ae*. *aegypti* males with female mosquitoes. A wood floor was added to the structure to facilitate detecting dead mosquitoes on it; the greenhouse was at the Centro de Biotecnología Genómica, Instituto Politécnico Nacional, Reynosa, Mexico.

For each replicate, a single male *Ae*. *aegypti* (FEM or uninfected) was confined with 20 females in a small greenhouse constructed of gauze and cotton walls (6 x 3 x 2.80 meters) (Fig 2). This experiment examined the ability of FEMs to search for female mates in a large space and compared the copulation activity with that of an uninfected male. As with the laboratory experiments, the male (FEM or uninfected) and female mosquitoes were confined from 16:00 h to 19:00 h after which all of the mosquitoes were captured with a mouth aspirator. The females were then inspected with a UV light for florescent powder and the spermathecae were dissected to record insemination. First, 10 replicates conducted over 10 sequential days were carried out for uninfected males (yellow powder). After 5 days, the same process was carried out for FEMs (red powder). In contrast to the laboratory assay, 5 categories of the nominal RV were recorded: Inseminated by FEM, mating attempts made by FEM (where female remains uninseminated), inseminated by uninfected male, mating attempts by uninfected male, and uninseminated females not contacted by any male. A digital higrothermometer was placed into the greenhouse to register both temperature and relative humidity (RH) during the 3-hour interval of activity of the released males.

### Effect of *M*. *anisopliae* on the successive copulation time in *Ae*. *aegypti* males

For each replicate, 20 male *Ae*. *aegypti* mosquitoes were exposed 24 hrs, either to fungus (3 replicates) or a clean control filter (3 replicates). Immediately after exposure to treated or clean filters, the 20 males per replicate were individually isolated in 1-liter translucent plastic bottles covered with a mesh and a pad soaked in 5% sucrose. Then, after 30 minutes of rest, 5 virgin female mosquitoes were confined successively with the same male. For each male, observations began at 9:00 am and concluded when he copulated with the 5^th^ female (a process completed in less than 4 hours in the experiment). Here, “copulation” was the direct genital contact of a couple that lasted at least for 10 seconds [21]. Immediately after copulation the female was removed and replaced by a new one with a mouth aspirator. For all replicates the same 2 people measured the time to each copulation using chronometers. The total time (minutes) for each *Ae*. *aegypti* male to copulate successively with 5 different female mosquitoes was the average of two observations. So, the RV registered here was total time as the minutes elapsed since the introduction of the 1^st^ female up to the copulation with the 5^th^ one.

### Sperm production of *M*. *anisopliae*-exposed *Ae*. *aegypti* males

The sperm production in 3-day old male *Ae*. *aegypti* after exposure to *M*. *anisopliae* was examined. Two treatments were tested: males exposed for 24-h to the ED, plus a control where the males were exposed to clean filters for 24-h. Each treatment had 3 replicates. Initially for each replicate (FEM, control), approximately 600 eggs (taken from different cages) were placed in 3 enamel trays with 1 liter of distilled water. After 24-h, 300 first instar larvae were transferred to a tray (50 x 50 x 10 cm) with 5 liters of distilled water (60 larvae/liter) and fed daily with 1.5 g of dog food. Then, to acquire mainly male pupae, 100 small pupae within the first 24-h of pupation per tray were transferred to 1-liter plastic cup, which was placed in a cage for adult emergence with a pad soaked in 5% sucrose. Any females that emerged were removed, and when the males were 2-day old, they were exposed to the fungus or control filters. Starting the following day (3-d old), 10 males mosquitoes were removed for dissection each day for 5 consecutive days per day (n = 50). The males were immobilized by exposure to 4°C for 15 minutes and the spermatozoa were counted [27]. Testis and seminal vesicle were dissected gently, placed in a culture multi-well plate with 50 μL of phosphate-buffered saline (PBS), and added 150 μL of PBS to have 200 μL of stock solution per male per well. From each well, 5 μL was extracted and deposited in a concave microscope slide. Slides were dried at laboratory conditions (23 ± 2°C, 60 ± 10% RH) for 1-h, washed in 70% ethanol, stained with Giemsa for 1-h, washed 5 times in 1 ml of distilled water and again dried at laboratory conditions. Spermatozoa heads stained red were counted under a 40x microscope and multiplied by 40 to convert the spermatozoa number observed in 5 μL to the total number in 200 μL of stock solution per male.

### Estimation of conidia load in fungus-exposed males and copulated female *Ae*. *aegypti*

To measure the conidial load attached to the body of male mosquitoes, 200 males earlier exposed to the ED for 24-h were placed in a 5-liter plastic flask covered with a mesh and then killed immediately by exposure to -20°C for 3 minutes. Next, 20 pools of 3 males (n = 180) each placed in 1.5 mL centrifuge tubes containing 500 μL of the same mixture used for conidia harvest. Three replicates were carried out for a total of 60 pools of 3 males each. Conidia were removed from the cuticle of the 3 males per tube by vortexing 3 times for 1 second, the mosquitoes were discarded, and each tube containing the conidia was centrifuged at 5,000 rpm for 5 minutes. The pellet with conidia was re-suspended in 20% lactophenol-blue solution [28] and 3 conidia counts by hemocytometer were used to determine the mean ± SE of conidia mL^-1^ per pool. Likewise, a scanning electron microscope (SEM) was used to snap the conidia attached to the cuticle of front tarsi of 10 fungus-contaminated males prepared as described previously [29]. Males were killed by freezing, dissected gently, and mounted on metal stubs, then glued with copper paint, gold-coated, and examined through the SEM.

The conidial load delivered to female mosquitoes during the 1^st^ and 5^th^ successive copulation was compared between females copulating with FEMs (males exposed to fungus for 24h) or uninfected males exposed to clean filters. Three replicates of 20 males each were carried out. Each replicate was contained in a 1-liter transparent plastic bottle covered with a mesh and a pad soaked in 5% sucrose. Individual males were introduced into the experimental chamber, and then after 30 minutes of rest, 5 virgin female mosquitoes per treatment were confined successively with the same male. After copulation, each female was removed promptly with a mouth aspirator, and a 2^nd^one was introduced into the flask to induce copulation, and the process repeated until the 5^th^ female was introduced and copulated by the same male. Afterwards, the conidial load adhered to the bodies of the females that participated in the 1^st^ and 5^th^ copulations was estimated on the same day as described for males above.

### Survival of *Ae*. *aegypti* females exposed to topical application of *M*. *anisopliae*

The survival of females after exposure to 5,000 conidia mL^-1^ by topical application of 300 μL per insect was assessed. This dosage was chosen to represent the quantity of conidia that FEMs are able to transfer to females during the 5^th^ copulation (see Results). Two treatments were tested: females that received on the thorax 300 μL of a stock of 5000 conidia mL^-1^ and those females that received the same volume of the mixture used to yield conidia but free of fungus. Each treatment had 3 replicates and 10 females per replicate; mortality was registered daily up to the death of the last female while cadavers and sporulation were processed as described above.

### Statistical analysis

For survival, the median lethal time (LT_50_) in each treatment was computed by the Kaplan-Meier model and compared by a χ^2^ log rank test; the survival of treated and uninfected individuals was compared separately for males and females. To compare copulation activity between FEMs and control males in the laboratory, the following model was constructed: Females = treatment (inseminated by FEM, inseminated by male, inseminated by both, not inseminated) + day + treatment*day + error, which measured the variation of the least square means (LSMs) of female mosquitoes among treatments for the entire set of 10 days, within each day, and the interaction between treatment and day. For the green-house experiments, the variation of the LSMs of the RV was analyzed among treatments and copulation status by the model: Females = treatment + copulation status + error, where “copulation status” was defined as a group of females with presence or absence of insemination/*M*. *anisopliae*-infection. To assess the effect of *M*. *anisopliae* on the time (minutes) required by a male to copulate 5 successive female mosquitoes, a dataset was constructed for total time for the 2 treatments (24-h and control). Sixty data points were gathered per treatment and 120 data points for the full dataset. The LSM of “minutes” per male was examined among treatments and replicates with the model: Minutes = treatment + replicate + error. For the sperm production, the LSMs for treatments (24-h and control) were computed from the counts of spermatozoa per male, and compared between treatments for the 5 days, among days within the same treatment and for the interaction treatment*day by the model: Females = treatment + day + treatment*day + error. To examine the conidial loads, the arithmetic means (rounded to the nearest digit) from 3-mosquito pools of the load of males, the load of females of the 1^st^ and the 5^th^ copulation, were used as data to estimate and compare the LSMs by the model: Load = treatment + copulation + pool + error.

All the models were negative binomial (NB) regressions ran by the procedure glimmix with SAS 9.4, a method that computed the LSMs and ran F and t tests by Tukey-Cramer multiple comparisons to measure the variation of LSMs among the explanatory variables (qualitative and quantitative). The procedure also estimated the robustness of each model by the goodness of fit of the RV to the NB distribution by the ratio Pearson χ^2^ / freedom degrees (total observations), which should be ≤ 1 [30–31].

## Results

### Survival of *Ae*. *aegypti* males and females exposed to *M*. *anisopliae*

All *Ae*. *aegypti* male mosquitoes died within 6 days post-exposure (PE), whereas survival in the control extended beyond 30 days. The LT_50_ for FEMs was 3.69 ± 0.16 days compared to 23.62 ± 0.58 days for uninfected males (χ^2^ = 168.96, df = 1, p < 0.0001). The sporulation rate in cadavers of FEMs was 100% indicating that all *Ae*. *aegypti* males deaths were indeed caused by fungal infection. The survival of female mosquitoes exposed to 5,000 conidia mL^-1^ by topical application was significantly shorter (LT_50_ = 3.36 ± 0.25 days) than for uninfected females (LT_50_ = 25.80 ± 0.60 days; χ^2^ = 65.06, df = 1, p < 0.0001).

### Effect of *M*. *anisopliae* infection on the mating activity of male *Ae*. *aegypti* under laboratory and small greenhouse conditions

**Fig 3:**
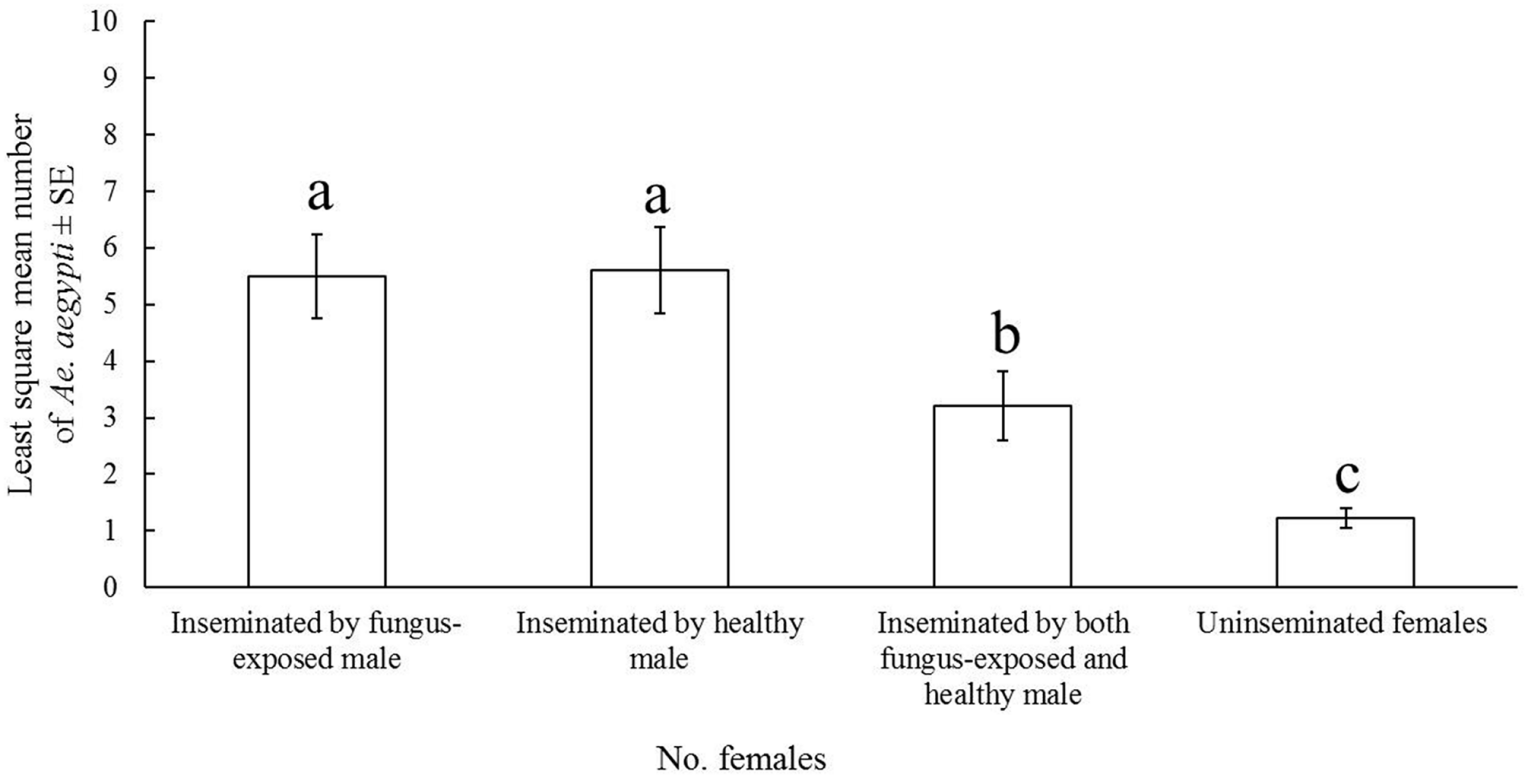
Daily number of *Ae*. *aegypti* females mated by a *M*. *anisopliae*-exposed male and an uninfected one in laboratory. Data is the least square means (LSMs) ± standard error (SE) number of *Ae*. *aegypti* female mosquitoes in each insemination class calculated with a glimmix model from 10 replicates. Different letters above bars denote significant differences (p < 0.05) accordingly to by pair-wise Student *t* tests ran by Tukey-Cramer multiple comparisons.

In the laboratory, there was no difference in the ability of FEMs to find and copulate with confined females with that of uninfected males. The LSM number of inseminated females was 5.47 ± 0.75 for FEMs, and 5.59 ± 0.76 for uninfected males. Interestingly, 3.21 ± 0.61 females had and just one female had mixed inseminations whereas only 1.22 ± 0.17 females had no evidence of copulation (Fig 3). The LSMs of female mosquitoes were different only for the explanatory variable “treatment” in the model (F = 24.93, df = 3, p< 0.001) meanwhile day and interaction treatment*day were not significant. The model was reliable; the NB goodness of fit test showed a ratio Pearson χ^2^/freedom degrees of 0.58.

**Fig 4:**
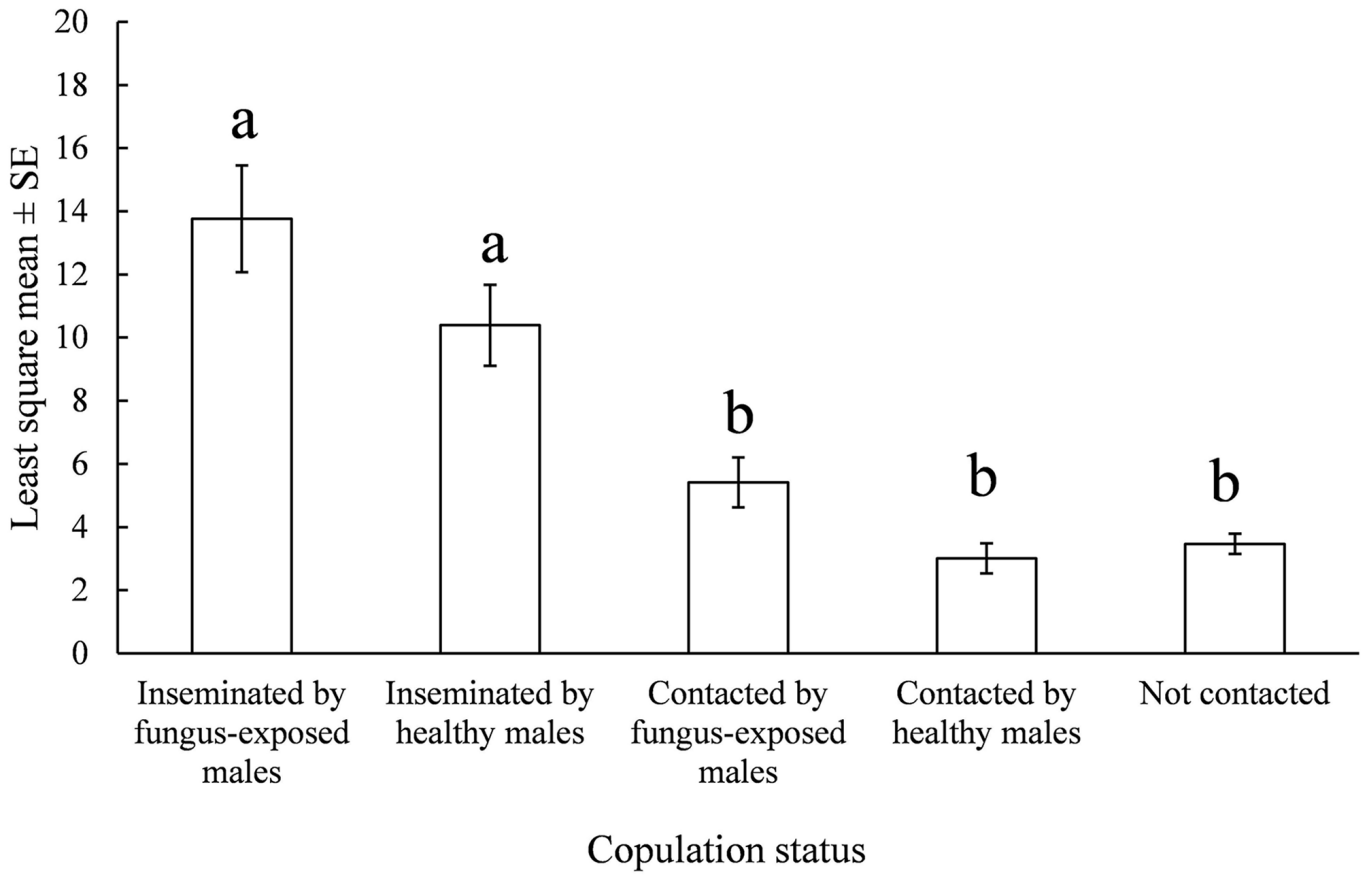
Daily number of *Ae*. *aegypti* female mosquitoes mated by a fungus-exposed and an uninfected male in a greenhouse. Data are the least square means (LSMs) ± standard error (SE) number of *Ae*. *aegypti* female mosquitoes in five “copulation status” (combination of insemination or not/*M*. *anisopliae*-infection). LSMs were calculated by a glimmix model from 10 replicates. Different letters above bars denote significant differences (p < 0.05) accordingly to pair-wise *t* tests conducted by Tukey-Cramer multiple comparisons.

In the small greenhouse, there was no difference in the ability of FEMs and uninfected males to search for and contact females. The LSMs number of females were not affected by treatment and day, but only by copulation status (F = 9.31, df = 4, p<0.0001) in the model, which was robust with a ratio Pearson χ^2^/ freedom degrees of 0.69; that is there was no statistical difference between the average number females inseminated by FEMs (9.86 ± 1.44) or controls (8.17 ± 1.17). Furthermore the number of females that were grasped (marked with red powder) but not inseminated (copulation attempts) by FEMs (7.48 ± 1.18) was greater than controls (with yellow powder) captured by uninfected males (3.25 ± 0.62); though both groups did not differ from total females (with no powder marking) that were not contacted by any male (5.10 ± 0.50) (Fig 4). During this experiment the daily temperature varied between 28°C and 35°C, and RH between 68 and 88%.

### Effect of *M*. *anisopliae* on the successive copulation time in *Ae*. *aegypti* males

**Fig 5:**
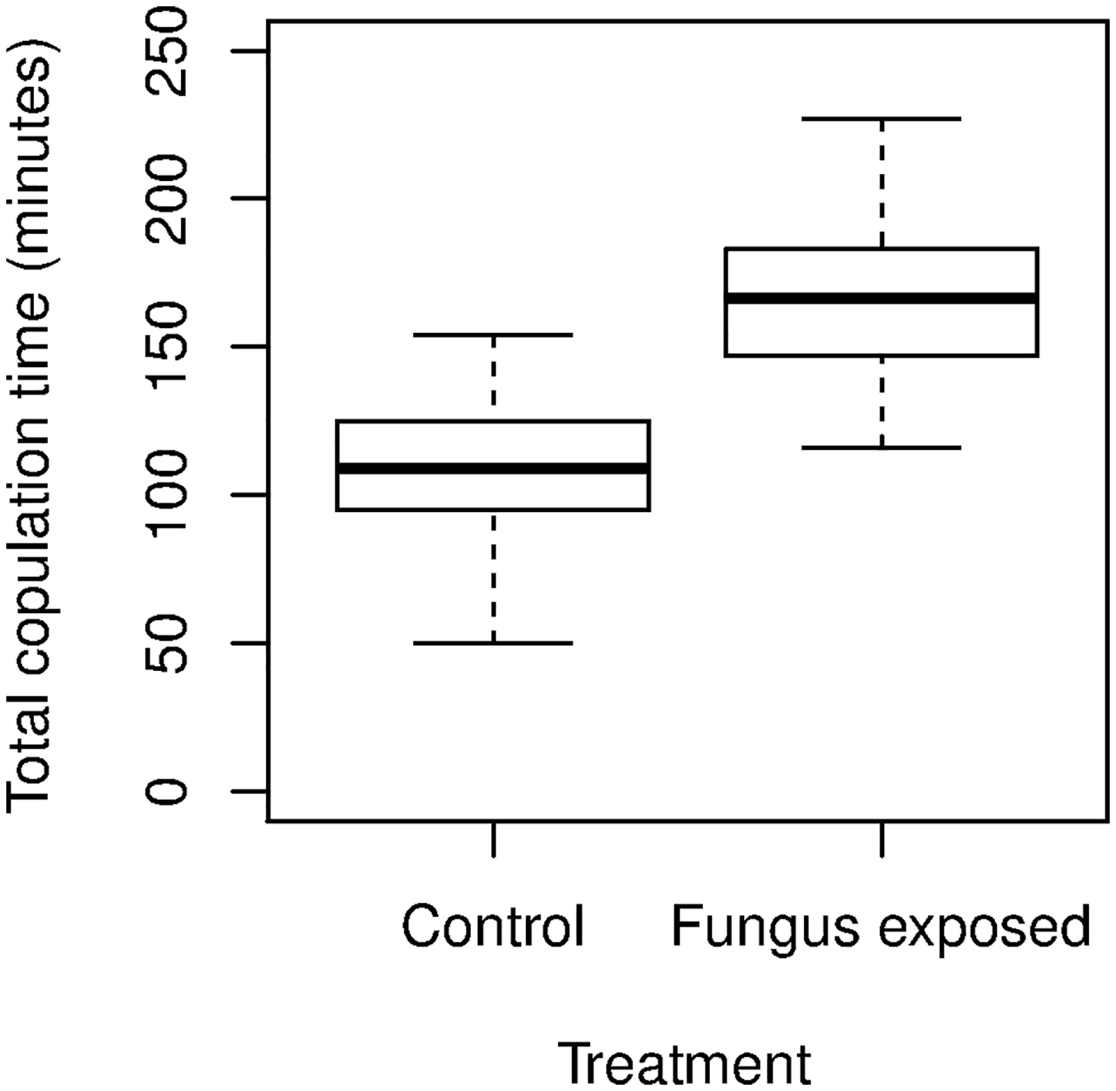
Comparison of the total time taken for one *Ae*. *aegypti* male to successively copulate with 5 females. For treatment (n = 3 replicates), the male mosquitoes were exposed to *M*. *anisopliae* (5.96 x 10^7^ conidia cm^-2^) for 24-h, and control males were exposed to untreated filter papers for 24-h.

The total time (minutes) required by males to copulate with 5 successive female mosquitoes varied between treatments (F = 97.36, df = 1, p< 0.0001). The ratio Pearson χ^2^/freedom degrees of 0.98 indicated an acceptable NB goodness of fit. Therefore, the LSMs for 24-h exposure and control were 165.61 ± 3.58 and 109.23 ± 2.80, respectively. The total time invested by FEMs was 34% longer than that of uninfected males. For FEMs the total time to attempt or to successfully copulate ranged between 110 and 220 minutes PE, whereas for uninfected males the range was 50 to 150 minutes PE (Fig 5).

### Sperm production of *M*. *anisopliae*-exposed *Ae*. *aegypti* males

The LSM of spermatozoa per male differed between treatments (F = 1015.31, df = 1, p< 0.0001), among days (F = 12.42, df = 4, p< 0.0001), and in the interaction treatment*day (F = 167.85, df = 8, p< 0.0001). The ratio Pearson χ^2^/ degrees of freedom of the NB goodness of fit test was 0.97. On day 1 PE, the LSM of spermatozoa per male after 24-h fungus exposure was 1,029 ± 49, which was 46% less than the 1,913 ± 36 spermatozoa in uninfected males. At day 5 PE, the LSM was 550 ± 30 spermatozoa per FEM, which was 92% less than the spermatozoa of uninfected males that had increased up to 6,601 ± 16 per male. Across the entire 5 day evaluation, sperm production in FEMs was reduced by 47% but was augmented by 71% in uninfected males.

### Estimation of conidia load in fungus-exposed male and copulated female *Ae*. *aegypti*

**Fig 6:**
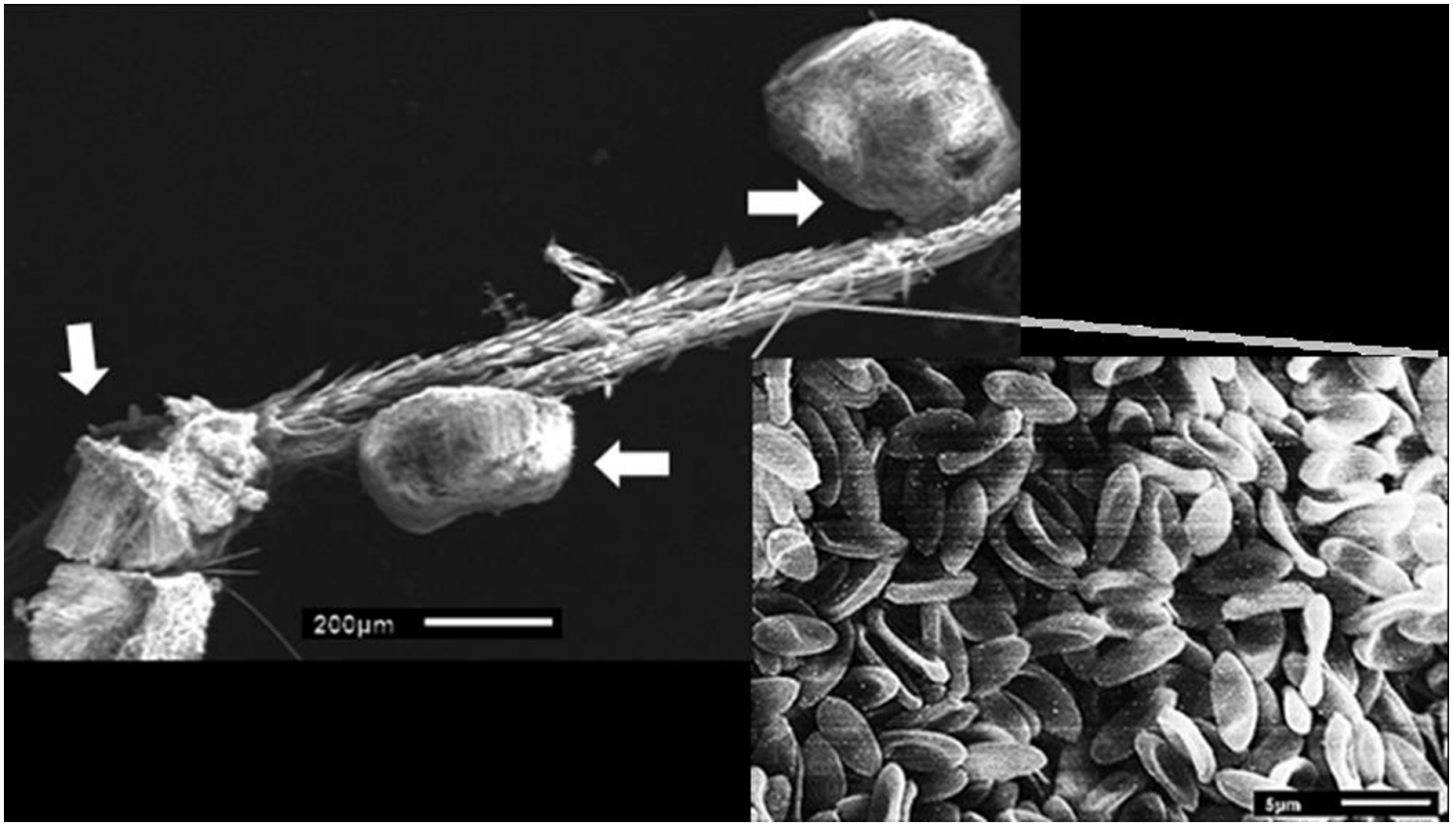
*M*.*anisopliae* conidia attached to front tarsal segments of a male of *Ae*. *aegypti*. Intersection of gray line denotes the conidia layer pasted on the tarsi and white arrows show the polyhedronic shapes of conidia clusters which appeared pasted on tarsal segments.

The LSM of conidia per pool varied significantly between treatments (F = 63.51, df = 2, p< 0.0001) in the model. The ratio Pearson χ^2^/freedom degrees of the NB goodness of fit test was 0.29. The LSM estimated per pool of three FEMs was 147,866± 21,064 mL^-1^, equivalent to 49,288 ±7,021 mL^-1^ per individual FEM. The SEM photograph indicated that conidia layers and clumps of polyhedronic shape of conidia remained attached on cuticle of front tarsi (Fig 6). The LSM conidial load per pool of 3 females from those that participated in the 1^st^ copulation was of 31,348 ± 4,507 spores mL^-1^(10,449 ± 1,502 mL^-1^ per individual female), whilst the same for those involved in the 5^th^ copulation was 15,811 ± 2,285 conidia mL^-1^(5,270 ± 761 mL^-1^ per individual female). Therefore, the conidial load of females of the 5^th^ copulation was 50% lower than for females from the 1^st^ copulation, and only 10% of the conidial load of males.

## Discussion

Regarding direct impacts on survival, the *M*. *anisopliae* strain (CBG-Ma2) had a LT_50_ of 4 days, which was similar to that reported for other strains [7–8, 10]. Although it is difficult to compare results across different studies, here the highest copulation rate (inseminated and not) of 75% recorded for FEMs is comparable to the 65%–85% range reported when 5 males were confined for 24 hours with 20 females in large field cages [32]. Concerning the parameters pertaining to mating activity, both negative and beneficial results were recorded. The negative impacts were that a longer time was invested by FEMs to successively copulate with 5 females than uninfected males. Also fungus infection reduced the sperm production by 87% by day 5 PE.

It is well established that following six inseminations in rapid succession, sperm becomes depleted in *Ae*. *aegypti* males, and they require 3 days of “sexual resting” to replenish the seminal vesicles [22–33]. Therefore our results suggest that if FEMs are released in field, they will only spread the pathogen during their first swarming event, but this should subsequently result in decreased oviposition by exposed females.

Fungus infection, however did have impacts that were beneficial for the potential use of auto-dissemination as a biocontrol tool. There was no difference in the ability of FEMs to find and copulate with females in the laboratory or small greenhouse when compared with uninfected males. More interestingly, in the small greenhouse the FEMs made more mating attempts without insemination than the uninfected males. This difference indicates that the fungus increases male copulations by 27%, which would facilitate auto-dissemination control strategies. The reason for this observed difference however, is difficult to explain. One hypothesis is this increase in mating attempts is an indirect effect associated with the innate immune response of male *Ae*. *aegypti*. It is possible that the fungus modulates male sexual behavior to increase its dispersal and propagation, a phenomenon noted in other fungus/insect interactions [34].

The tarsal contact occurring during attempted and successful copulations clearly transfers conidia from the FEMs to females. When an *Ae*. *aegypti* male copulates, he uses his front tarsi to hold the female’s hind femora and other legs [21, 25]; and with our ED and exposure methodology we observed the *M*. *anisopliae* conidial loads primarily on the legs of the FEMs (Fig 6). The 5^th^ female which was copulated received a load of 5,200 conidia mL^-1^ which was 10% of that on the males (49,000 mL^-1^). Further, through direct topical application, we observed that this quantity of conidia was sufficient (LT_50_ = 3 days) to cause significant mortality of the females. This is supported by a recent study in which *Ae*. *aegypti* females infected with Indian strains of *M*. *anisopliae*, there was a mean lethal concentration (LC_50_) of 5,920conidia mL^-1^[35].

In conclusion, our study is the first to measure the effect of *M*. *anisopliae* on copulation behavior of male *Ae*. *aegypti*. Although the fungus killed the 50% of males in 4 days, the FEMs in semi-field conditions captured up to 15 females in successful copulations (8 with insemination) or mating attempts (7 with no insemination) during the first 3-h of confinement/release, and a single FEM transferred significant and lethal amounts of conidia to the first 5 females copulated successively. Our baseline results suggest that biological control of *Ae*. *aegypti* by releasing *M*. *anisopliae*-contaminated males to spread the pathogen by mating with wild females is feasible.
